# Mental health assessment in health checks of participants aged 30–49 years: A large-scale cohort study

**DOI:** 10.1016/j.pmedr.2017.12.011

**Published:** 2018-01-05

**Authors:** Christine Geyti, Helle Terkildsen Maindal, Else-Marie Dalsgaard, Kaj Sparle Christensen, Annelli Sandbæk

**Affiliations:** a Research Unit for General Practice & Section for General Medical Practice, Department of Public Health, Aarhus University, Bartholins Allé 2, 8000 Aarhus, Denmark; b Section for Health Promotion and Health Services Research, Department of Public Health, Aarhus University, Bartholins Allé 2, 8000 Aarhus, Denmark; c Steno Diabetes Centre Copenhagen, Health Promotion Research, Niels Steensens Vej 2, 2820 Gentofte, Denmark

**Keywords:** Mental health, General practice, Primary health care, Health promotion, Preventive health services

## Abstract

Mental distress is an independent risk factor for illness related impairment. Awareness of mental health (MH) allows prevention, but early detection is not routinely performed in primary care. This cohort study incorporated MH assessment in a health promoting programme. We described the level of poor MH among health check participants, explored the potential for early intervention, and the potential for reducing social inequality in MH. The study was based on 9767 randomly selected citizens aged 30–49 years invited to a health check in Denmark in 2012–14. A total of 4871 (50%) were included; 49% were men. Poor MH was defined as a mental component summary score of ≤ 35.76 in the SF-12 Health Survey. Data was obtained from national health registers and health check. Participants with poor MH (9%) were more socioeconomic disadvantaged and had poorer health than those with better MH. Two thirds of men (64%) and half of women (50%) with poor MH had not received MH care one year before the health check. Among those with (presumably) unrecognized MH problems, the proportion of participants with disadvantaged socioeconomic characteristics was high (43–55%). Four out of five of those with apparently unacknowledged poor MH had seen their GP only once or not at all during the one year before the health check. In conclusion, MH assessment in health check may help identify yet undiscovered MH problems.

## Highlights

• Nine % of health check participants were identified with poor mental health (MH).
• Poor MH was associated with lower socioeconomic status and worse health.
• Only 45% of persons with poor MH had received MH care.
• High proportion of low socioeconomic status among those who had not received MH care
• MH assessment in health checks may help identify yet undiscovered MH problems.

## 1 Introduction

Poor mental health (MH) is a growing public health concern with considerable human, social, and economic costs due to its correlation with mortality (Christensen et al., 2017), physical comorbidity (Dong et al., 2012, Mezuk et al., 2008, Prince et al., 2007, Gunn et al., 2012), socioeconomic deprivation (Korkeila et al., 2003, Kuruvilla and Jacob, 2007, Gunn et al., 2008), unhealthy behaviour (Hamer et al., 2009, Pisinger et al., 2009), and poor quality of life (Moussavi et al., 2007). Moreover, social inequality in MH is evident (Pinto-Meza et al., 2013). The risk of poor MH peaks during early- to mid-life (Kessler et al., 2007), and mental illness is one of the leading causes of disability in this age span (Murray et al., 2012). The prevalence of poor MH among Danish adults is 10% (Christensen et al., 2014). Early detection of poor MH is essential to improve both mental and physical health status and to prevent development of manifest mental disease (World Health Organization, 2004). Despite promotion of MH as a key priority for public health policy in Europe (Wahlbeck, 2011), real community-based collaboration and research on MH promotion in a primary care setting are sparse (Fernandez et al., 2015).

Routinely offered health checks have been proposed as a means to improve the public health (Cochrane et al., 2012, Royal Australian College of General, 2012), but the effects and the optimal content remain to be determined. Few studies on health checks including MH assessment have, to our knowledge, been published (Bjerkeset et al., 2006, Crisp and Priest, 1971), and in those cases the purpose was to identify mental disorders. However, MH ought to be considered as a broader concept than merely the presence or absence of mental disorders. The World Health Organization (WHO) defines good MH as ‘a state of well-being in which every individual realizes his or her own potential, can cope with the normal stresses of life, can work productively and fruitfully, and is able to make a contribution to her or his community’ (World Health Organization, 2001). If the means of MH assessment is to improve MH, and not only detect mental disorders, a generic measure of poor MH may be required.

In a cohort study, we evaluated the use of the Mental Component Summary (MCS) of SF-12 in a community-based health check with close links to primary care. Overall, we aimed to investigate the level of poor MH among health check participants aged 30–49 years, the potential for early intervention, and the potential for reducing social inequality in MH. Based on literature on poor MH in the Danish general population (Christensen et al., 2014) we hypothesized that poor MH among health check participants would be associated with disadvantaged socioeconomic characteristics, health behaviour, and health status. We further expected a higher proportion of participants with disadvantaged than of advantaged socioeconomic characteristics among those with presumably undetected poor MH (Packness et al., 2017). The objectives were (i) to describe associations between poor MH and socioeconomic characteristics, health behaviour, and health status among participants in a general health check, and (ii) to describe the socioeconomic characteristics of participants with presumably yet undetected poor MH.

## 2 Methods

### 2.1 Study design and population

The *Check Your Health* health promotion programme (Maindal et al., 2014) features a population-based preventive health check in the local health centre followed by a face-to-face consultation with the person's general practitioner (GP). The health check is offered to all citizens aged 30–49 years in Randers Municipality, Denmark in 2012–2017. The *Check Your Health* health promotion programme aimed at this age range because of the potential for prevention of development of both mental and physical diseases and possible complications. The health check focuses on risk factors for long-term conditions, e.g. cardiovascular disease (CVD) and diabetes, and on MH.

**Fig. 1:**
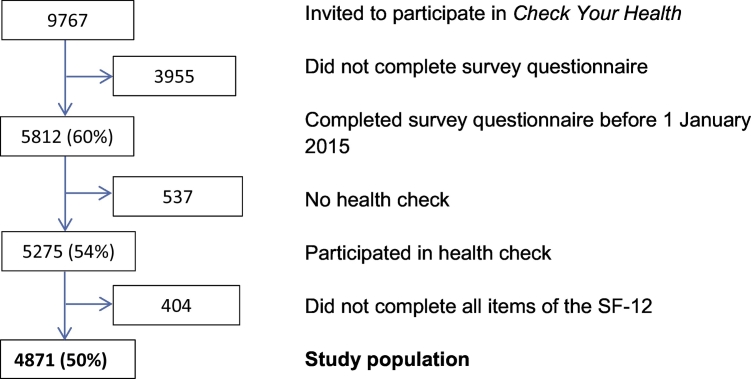
Flowchart of inclusion of participants aged 30–49 years from Randers Municipality, Denmark, in the *Check Your Health* preventive programme from April 2012 to October 2014. SF-12: 12-item Short Form Health Survey, version 2.

Except patients with terminal illness, all citizens in Randers Municipality at the age of 30–49 years at January 1, 2012 were randomised to an individual invitation date to the *Check Your Health* programme (n = 26,216). Citizens who were invited within the first approximately 2½ years (18 April 2012 to 1 October 2014), n = 9767, were eligible for the present retrospective cohort study. In the cohort, we included participants in the health check examination who completed a survey on MH (Fig. 1). A total of 4871 were included in the cohort (50% of the invited); men composed 49% of the study population. Informed consent was obtained from all participants in the study. The study was approved by the Danish Data Protection Agency. Approvement from The National Committee on Health Research Ethics was not required since the study used data from the ongoing *Check Your Health* programme.

### 2.2 Data sources

Survey data and clinical data were obtained from *Check Your Health* and linked to Danish registers through each participant's unique personal identification number, which is assigned to all persons with permanent residence in Denmark (Pedersen, 2011).

#### 2.2.1 Mental health

MH was measured by the Mental Component Summary (MCS) score of the validated Danish version of the 12-item Short Form Health Survey (SF-12), version 2 (Gandek et al., 1998, Ware et al., 1996, Ware et al., 2002). MSC is based on 12 items on general self-rated health, mood and anxiety symptoms, physical health, and functional limitations during the past four weeks (Ware et al., 1996). For each item there are three to five response options (e.g. ‘all/most/some/a little/none of the time’). Standard general population norms and scoring algorithm (US norms of 1998) were used to calculate MCS score (Ware et al., 2002). Calculation of MCS score is dependent on full completion of SF-12. The MCS score is measured on a continuous scale between 0 and 100; higher score reflects better MH. MCS score was categorised into poor (≤ 35.76), good (≥ 48.26), and moderate MH (in between) based on a Danish national health survey (Christensen et al., 2010). Rather than targeting specific psychiatric diagnoses, MCS provides a generic measure of MH. However, MCS is also validated against diagnoses of mental disorders (Vilagut et al., 2013, Kiely and Butterworth, 2015, Gill et al., 2007). A cut-point of ≤ 36 has a sensitivity of 0.62 for 30-day diagnosis of any depressive disorder and a sensitivity of 0.73 for 30-day generalized anxiety disorder (Kiely and Butterworth, 2015). The corresponding specificities are 0.88 and 0.90 (Kiely and Butterworth, 2015).

#### 2.2.2 Mental health care

MH care was defined as at least one of the following within a year before completing the survey questionnaire: psychometric test or talk therapy by GP, contact to psychologist or psychiatrist, or psychotropic medication as recorded in the Danish national health registers (Table 1). These will hereafter be referred to collectively as ‘MH care’.

**Table 1:** Mental health care one year before health check.

Variable	National health registers	Notes
Psychometric test by GP	NHSR	Approved psychometric tests, e.g. diagnostic tests for depression or anxiety.
Talk therapy by GP	NHSR	By GPs under psychological supervision. Max. 7 sessions per year for each patient.
Contact to psychologist	NHSR	After referral from GP
Psychotropic medication	DNPR	Redeemed prescription of the following medication (ACT codes): antipsychotics (N05A), anxiolytics (N05B), hypnotics and sedatives (N05C), antidepressants (N06A), psychostimulant medication (N06B), anti-dementia drugs (N06D).
Contact to psychiatrist	NHSR NPR	Private psychiatrists Psychiatric hospitals (inpatients and outpatients)

ACT: Anatomical Therapeutic Chemical Classification. NHSR: Danish National Health Service Register (Andersen et al., 2011). DNPR: Danish National Prescription Register (Kildemoes et al., 2011). NPR: Danish National Patient Register (Lynge et al., 2011).

#### 2.2.3 Health behaviour and physical health

Data on smoking, alcohol risk behaviour, and self-rated health was collected from the health check survey. From April 2012 to July 2013, alcohol risk behaviour was calculated by CAGE-C and defined as > 2 positive answers to items 1–4 and 6, or one positive answer to items 1–4 and 6 plus alcohol intake on > 4 days per week (Zierau et al., 2005). From August 2013 to October 2014, alcohol risk behaviour was calculated by AUDIT (Saunders et al., 1993) and defined as ≥ 8 points for women or ≥ 8 points plus alcohol intake ≥ 2 times per week for men. Self-rated health was categorised into good and fair/poor measured by the SF-12 (item 1: In general, would you say your health is…) (Ware et al., 1996).

Height and weight were measured at the health check, and body mass index (BMI) was categorised according to standard cut-off points into < 18.5, 18.5–24.9 kg/m^2^, 25–29.9 kg/m^2^, and ≥ 30 kg/m^2^ (World Health Organization - Europe, 2017). Since only 30 participants had a BMI < 18.5 kg/m^2^, the two lower categories were collapsed. Blood pressure was measured with Omron M6, Omron Healthcare Europe B.V. Cholesterol and low-density lipoprotein (LDL) was measured by finger blood test with Alere Cholestech LDX System, Alere Denmark. Glycated haemoglobin (HbA1c) was measured by finger blood test with DCA Vantage Analyzer, Siemens Healthcare, Siemens AG, Germany. High risk of CVD was defined in line with the guidelines by the Danish Society of Cardiology (Danish Society of Cardiology, 2017a, Danish Society of Cardiology, 2017b) as at least of one of the following: systolic or diastolic blood pressure > 160/100 mm Hg, total cholesterol > 8 mmol/L, LDL > 6 mmol/L, HbA1c ≥ 48 mmol/mol, or 10-year risk of fatal CVD ≥ 5% based on gender, age, systolic blood pressure, total cholesterol, and smoking status (SCORE, low-risk chart) extrapolated to age 60 years (Perk et al., 2012). Trained healthcare staff performed the measurements by standardised methods, e.g. height measured at deep inspiration with heels touching wall; mean systolic and diastolic blood pressure from three measurements on left arm with 1 min intervals after 5 min rest.

Face-to-face contacts to GP, except pregnancy consultations, were obtained from the NHSR and were categorized into 0, 1, 2–4, or ≥ 5 contacts within a year before completing the survey questionnaire.

#### 2.2.4 Demographic and socio-economic characteristics

Demographic and socio-economic data for the year before invitation was obtained from administrative registers managed by Statistics Denmark (Pedersen, 2011, Statistics Denmark, 1991, Pedersen et al., 2011).

Country of origin was grouped into western or non-western countries (Statistics Denmark, 2016). Cohabitation was dichotomised into cohabitant (married or living with a partner) or living alone (including widows and divorced). Education was categorised into ≤ 10, 11–15, and > 15 years of education according to the International Standard Classification of Education by the United Nations' Educational, Scientific and Cultural Organization (UNESCO) (International Standard Classification of Education, 2012). Equivalence weighted household income was calculated as recommended by the Organization for Economic Co-operation and Development (OECD) (OECD, 2013) and categorised into tertiles. Employment was grouped into ‘employed’, ‘unemployed/benefits’ (unemployed at least half of the year, being on activation, or receive sickness benefit or parental benefit), and ‘social welfare recipients’ (receiving early disability pension or social security).

### 2.3 Statistical analyses

Prevalence was reported with 95% confidence interval (CI)) and compared using Chi-square tests. Logistic regression models were used for estimating odds ratios (OR) (with 95% CI) associated with poor MH (Table 3). Model 1 presents crude estimates. Model 2 presents estimates adjusted for all variables in Table 3, except alcohol risk behaviour due to a large number of missings. P-values of < 0.05 were considered as statistically significant. Cells containing less than five observations were reported as ‘< 5’, in line with the regulations of Statistics Denmark. The statistical analyses were performed using Stata, version 14.0 (StataCorp, College Station, Texas) on the available data only. Thus, missing data were not imputed.

## 3 Results

Poor MH was identified in 8.8% of the participants (Table 2). More women (11.0%) than men (6.6%) had poor MH (data not shown). One in four (27.3%) non-Western participant was identified with poor MH. Less than six % of participants with good self-rated general health had poor MH. Participants with poor MH were more than twice as likely to have visited their GP five times or more as participants with good MH (Table 2). Nine % of those with poor MH had not visited their GP at all within the year before the health check.

**Table 2:** Baseline characteristics of participants aged 30–49 years in *Check Your Health,* stratified by mental health status. Denmark 2012–2014.

	Poor mental health		Moderate mental health		Good mental health		Total	Missing
	n	% (95% CI)	n	% (95% CI)	n	% (95% CI)		
n (%)	431	8.8 (8.7–9.7)	1217	25.0 (23.8–26.2)	3223	66.2 (64.8–67.5)	4871	0
Demographic characteristics								
Sex								0
Men	159	6.6 (5.7–7.7)	557	23.1 (21.5–24.8)	1693	70.3 (68.4–72.0)	2409	
Women	272	11.0 (9.9–12.3)	660	26.8 (25.1–28.6)	1530	62.1 (60.2–64.0)	2462	
Age								0
30–34	61	10.3 (8.1–13.1)	172	29.2 (25.6–33.0)	357	60.5 (56.5–64.4)	590	
35–39	115	10.9 (9.1–12.9)	286	27.0 (24.4–30.0)	657	62.1 (59.1–65.0)	1058	
40–44	128	9.8 (8.3–11.5)	324	24.8 (22.5–27.2)	854	65.4 (62.8–68.0)	1306	
≥ 45	127	6.6 (5.3–7.8)	435	22.7 (20.9–24.6)	1355	70.7 (68.6–72.7)	1917	
Country of origin								< 5/4871
Western	370	8.0 (7.2–8.8)	1144	24.6 (23.4–25.8)	3139	67.5 66.1–68.8)	4653	
Non-Western	59	27.3 (21.7–33.7)	73	33.8 (27.8–40.4)	84	38.9 (32.6–45.6)	216	
Socioeconomic characteristics								
Living alone								< 5/4871
No	291	7.6 (6.8–8.4)	922	24.0 (22.6–25.3)	2636	68.5 (67.0–70.0)	3849	
Yes	140	13.7 (11.7–16.0)	294	28.8 (26.1–31.7)	586	57.5 (54.4–60.5)	1020	
Income								0
Low	210	16.4 (14.5–18.6)	398	31.2 (28.7–33.8)	669	52.4 (49.6–55.1)	1277	
Medium	122	7.2 (6.1–8.6)	407	24.1 (22.1–26.2)	1159	68.7 (66.4–70.8)	1688	
High	99	5.2 (4.3–6.3)	412	21.6 (19.8–23.5)	1395	73.2 (71.2–75.1)	1906	
Education (years)								67/4871
0–10	102	14.7 (12.3–17.6)	170	24.5 (21.5–27.9)	421	60.8 (57.1–64.3)	693	
11–15	187	7.6 (6.6–8.7)	604	24.5 (22.8–26.2)	1676	67.9 (66.1–69.8)	2467	
> 15	129	7.8 (6.6–9.3)	415	25.2 (23.2–27.4)	1100	66.9 (64.6–69.1)	1644	
Occupational status								81/4871
Employed	288	6.6 (5.9–7.4)	1043	24.1 (22.8–25.4)	3005	69.3 (67.9–70.7)	4336	
Unemployed/benefits	36	21.7 (16.0–28.7)	46	27.7 (21.4–35.1)	84	50.6 (43.0–58.2)	166	
Social welfare recipients	98	34.0 (28.8–39.7)	97	33.7 (28.4–39.4)	93	32.3 (27.1–38.0)	288	
Health								
Daily smoker								69/4871
No	299	7.6 (6.8–8.4)	949	24.1 (22.7–25.4)	2697	68.4 (66.9–69.8)	3945	
Yes	124	14.5 (12.3–17.0)	247	28.8 (25.9–32.0)	486	56.7 (53.4–60.0)	857	
Alcohol risk behaviour								1451/4871
No	216	7.1 (6.2–8.1)	725	23.9 (22.4–25.4)	2097	69.0 (67.4–70.6)	3038	
Yes	38	9.9 (7.3–13.4)	106	27.7 (23.4–32.5)	238	62.3 (57.3–67.0)	382	
Body Mass Index (kg/m^2^)								6/4871
< 25	188	9.7 (8.5–11.1)	458	23.7 (21.8–25.6)	1287	66.6 (64.4–68.7)	1933	
25–29.9	129	6.8 (5.8–8.1)	478	25.3 (23.4–27.3)	1280	67.8 (65.7–70.0)	1887	
≥ 30	114	10.9 (9.2–13.0)	281	26.9 (24.3–30.0)	650	62.2 (59.2–65.1)	1045	
Self-rated health								0
Good	258	5.9 (5.3–6.7)	1016	23.4 (22.1–24.6)	3074	70.7 (69.3–72.0)	4348	
Fair/poor	173	33.1 (29.2–37.2)	201	38.4 (34.3–42.7)	149	28.5 (24.8–32.5)	523	
High 10-year risk of fatal CVD								0
No	343	8.4 (7.6–9.2)	1043	25.6 (7.6–9.3)	2688	66.0 (64.5–67.4)	4074	
Yes	88	11.0 (9.0–13.4)	174	21.8 (19.1–24.8)	535	67.1 (63.8–70.3)	797	
Number of contacts to GP 1 year before health check								0
0	38	4.0 (2.9–5.4)	181	19.0 (16.6–21.6)	733	77.0 (74.2–79.6)	952	
1	55	5.6 (4.3–7.3)	210	21.5 (19.0–24.2)	711	72.8 (70.0–75.6)	976	
2–4	143	8.3 (7.1–9.7)	434	25.2 (23.2–27.3)	1147	66.5 (64.3–68.7)	1724	
≥ 5	195	16.0 (14.0–18.2)	392	32.2 (29.6–34.8)	632	51.8 (49.0–54.6)	1219	

Poor mental health: MCS score of ≤ 35.76; moderate mental health: MCS score of > 35.76 and < 48.26; good mental health: MCS score of ≥ 48.26 (from SF-12, v. 2, US norms of 1998). Alcohol risk behaviour measured with Cage-C (≥ 2 positive answers to items 1–4 and 6, or one positive answer to items 1–4 and 6 plus alcohol intake on ≥ 4 days per week) or AUDIT (≥ 8 points for women, ≥ 8 points in addition to alcohol intake ≥ 2 times per week for men). High risk of cardiovascular disease (CVD): systolic or diastolic blood pressure > 160/100 mm Hg, total-cholesterol > 8 mmol/L, low density lipoprotein (LDL) > 6 mmol/L, (HbA1c) ≥ 48 mmol/mol, or 10-year risk of fatal CVD > 5% (SCORE, low-risk chart) extrapolated to the age of 60 years. AUDIT: Alcohol Use Disorders Identification Test. BMI: Body Mass Index. Cage-C: Cut down, Annoyed, Guilty, Early-morning (Copenhagen). DBP: diastolic blood pressure. GP: General practitioner. HbA1c: glycated haemoglobin. LDL: low-density lipoprotein. MCS: Mental component summary. SCORE: Systematic Coronary Risk Evaluation. SDP: systolic blood pressure. SF-12: 12-item short-form Health Survey.

Female sex, age younger than 45 years, and non-Western country of origin were associated with poor MH (Table 3). The multivariate analysis did not alter these estimates. Poor MH was associated with socioeconomic disadvantage, although education lost its association in the multivariate analysis. Daily smoking, alcohol risk behaviour, and high 10-year risk of fatal CVD were all associated with poor MH. BMI according to overweight (25–29.9 kg/m^2^), but not obesity (≥ 30 kg/m^2^), was associated with lower odds for poor MH compared to BMI < 25 kg/m^2^. A sensitivity analysis including alcohol risk behaviour in the multivariate analysis, showed no major changes of direction or magnitude of associations, but confidence intervals widened (data not shown).

**Table 3:** Odds ratios (OR) for poor mental health among participants in *Check Your Health*, Denmark, 2012–2014.

	Model 1^a^	Model 2^b^
Variables	OR (95% CI)	OR (95% CI)
Demographic variables		
Sex		
Men	0.57 (0.46–0.70)	0.60 (0.48–0.76)
Women	1	1
Age		
30–34	1.63 (1.18–2.24)	1.92 (1.36–2.72)
35–39	1.72 (1.32–2.24)	1.88 (1.41–2.52)
40–44	1.53 (1.19–1.98)	1.50 (1.13–1.98)
≥ 45	1	1
Country of origin		
Western	1	1
Non-Western	4.35 (3.17–5.98)	3.65 (2.49–5.35)
Socioeconomic variables		
Living alone		
No	1	1
Yes	1.95 (1.57–2.41)	1.42 (1.11–1.83)
Education (years)		
0–10	2.03 (1.54–2.67)	1.05 (0.75–1.48)
11–15	0.96 (0.76–1.22)	0.87 (0.67–1.11)
> 15	1	1
Occupational status		
Employed	1	1
Unemployed/benefits	3.89 (2.64–5.74)	2.93 (1.94–4.41)
Social welfare recipients	7.25 (5.53–9.51)	5.16 (3.70–7.22)
Health		
Daily smoker		
No	1	1
Yes	2.06 (1.65–2.58)	1.48 (1.14–1.92)
Alcohol risk behaviour		
No	1	1
Yes	1.44 (1.00–2.07)	1.75 (1.17–2.61)
Body Mass Index (kg/m^2^)		
< 25	1	1
25–29.9	0.68 (0.54–0.86)	0.73 (0.57–0.95)
≥ 30	1.14 (0.89–1.45)	0.93 (0.70–1.23)
High 10-year risk of fatal CVD		
No	1	1
Yes	1.35 (1.05–1.73)	1.31 (0.99–1.74)

a Crude ORs.

b Adjusted for all variables in Table 3 (N = 4658), except alcohol risk behaviour. Alcohol risk behaviour adjusted for all other variables in Table 3 (N = 3420).

### 3.1 Mental health care

More than half (55.5%) of participants (two in three men and half of women) with poor MH had not received any of the recorded MH care within the year before the health check (Table 4).

**Table 4:** Sociodemographic characteristics and health care utilization of participants aged 30–49 years identified with poor mental health in *Check Your Health*, stratified by having received mental health care prior to health check (no/yes). Denmark, 2012–2014.

	Mental health care					
	No		Yes		Total	Missing
	n	% (95% CI)	n	% (95% CI)	N	
n	239	55.5	192	44.5	431	0
Demographic characteristics						
Sex						0
Men	102	64.2 (56.3–71.3)	57	35.8 (28.7–43.7)	159	
Women	137	50.4 (44.4–56.3)	135	49.6 (43.7–55.6)	272	
Socioeconomic characteristics						
Living alone						0
No	162	55.7 (49.9–61.3)	129	44.3 (38.7–50.1)	291	
Yes	77	55.0 (46.6–63.1)	63	45.0 (36.9–53.4)	140	
Income tertile						0
Low	110	52.4 (45.6–59.1)	100	47.6 (40.9–54.4)	210	
Medium	68	55.7 (46.7–64.4)	54	44.3 (35.6–53.3)	122	
High	61	61.6 (54.5–70.8)	38	38.4 (29.2–48.5)	99	
Education (years)						13/431
0–10	51	50.0 (40.3–59.7)	51	50.0 (40.3–59.7)	102	
11–15	93	49.7 (42.6–56.9)	94	50.3 (43.1–57.4)	187	
> 15	87	67.4 (58.8–75.1)	42	32.6 (24.9–41.2)	129	
Occupational status						9/431
Employed	172	59.7 (53.9–65.3)	116	40.3 (34.7–46.1)	288	
Unemployed/benefits	19	52.8 (36.0–69.0)	17	47.2 (31.0–64.0)	36	
Social welfare recipients	42	42.9 (33.3–53.0)	56	57.1 (47.0–66.7)	98	
Healthcare utilization						
Number of GP contacts one year before health check						0
0	30	78.9 (62.4–89.5)	8	21.1 (10.5–37.6)	38	
1	43	78.2 (64.9–87.4)	12	21.8 (12.6–35.1)	55	
2–4	92	64.3 (56.1–71.8)	51	35.7 (28.2–43.9)	143	
≥ 5	74	37.9 (31.4–45.0)	121	62.1 (55.0–68.6)	195	

Mental health care: Psychometric test by GP, talk therapy by GP, contact to psychologist, contact to psychiatrist, or psychotropic medication. Poor mental health: MCS score ≤ 35.76 (SF-12, version 2, US norms of 1998). GP: General practitioner. MCS: Mental component summary. SF-12: 12-item short-form Health Survey.

The proportion of participants with poor MH who had not received MH care was lower in the most disadvantaged socioeconomic groups compared to the highest socioeconomic groups: Poor MH and no MH care was observed in 52.4% of the lowest income group vs. 61.6% of the highest income group; in 50.0% of those with 0–10 years of education vs. 67.4% of those with > 15 years of education; and in 42.9% of social welfare recipients vs. 59.7% of employed persons. No statistically significant differences was seen between participants living alone (55.0%) and cohabiting (55.7%). Four fifth of participants with poor MH who had one visit (or no visits) to the GP during the year before the health check had not received any MH care. Of participants with poor MH who were frequent GP visitors (≥ 5 visits), 38% had not received MH care.

## 4 Discussion

### 4.1 Main findings

We found that 8.8% of participants in the health check had poor MH. Poor MH was associated with low socioeconomic status (except education in the multivariate analysis), risky health behaviour, and increased risk of CVD. Two in three men and half of the women identified with poor MH had not received any of the investigated types of MH care as recorded in the national registers within the year before the health check. In contrast to what we expected, we found lower proportions of the most disadvantaged socioeconomic groups among those with (presumably) unrecognised MH problems. Still, around half of participants with poor MH from the most disadvantaged socioeconomic groups had not received MH care.

### 4.2 Strengths and limitations

A major strength of this large-scale population-based study is that it was implemented in the existing healthcare system. Thus we may have explored the realistic potential for further preventive interventions: The proportion of poor MH and the risk profiles of health check attenders are likely to be realistic, and the results are applicable for the development of future health initiatives in Denmark.

#### 4.2.1 Definition of poor mental health

Although SF-12 is widely used in population studies to estimate the MH status, there is no consensus on a cut-point for poor MH. The cut-point of ≤ 35.76 for MCS score in the present study was based on a Danish national health survey, where this definition corresponded to the 10% of the general population with worse MH (Christensen et al., 2010). With the previously mentioned relatively low sensitivities for depressive disorders and anxiety disorders (Kiely and Butterworth, 2015), one may argue that the cut-point may omit too many with psychiatric symptoms. However, the present study is not a population study that aims to assess the prevalence of any mental disorder in the population. It is rather an initiative that seeks to find clinically relevant cases in need of MH intervention. In addition, the cut-point seem appropriate for excluding persons without depressive or anxiety symptoms (Kiely and Butterworth, 2015), and including persons with severe psychiatric symptoms and/or severe impairment (Gill et al., 2007, Sanderson and Andrews, 2002). This suggests that participants identified with poor MH with the cut-point used in *Check Your Health* are likely to be in need of MH intervention. We do not argue that another cut-point may be more favourable. Yet, in consideration of the GPs' limited capacity to perform follow-up on risk patients from the health check, it would be advisable that the persons with the worst symptoms are prioritised first.

#### 4.2.2 Mental health care

A strength of the study is that we have complete data of all participants on the recorded MH care from national registers. The coverage of MH services is assumed to be good as the registers are used for reimbursement (Andersen et al., 2011), and registers have been shown to be better than surveys to inform on MH service utilization (Drapeau et al., 2011). A limitation is that the registers do not obtain information on informal or unrecorded MH care, such as counselling outside of the health services, visits to psychologist without referral from GP (for example self-financed or covered by private health insurance), talk therapy by the GP beyond seven consultations per year, or psychological support by GPs who do not have access to psychological supervision (Danish GPs under psychological supervision get additional reimbursement for up to seven talk therapy consultations per patient per year). The true proportion of MH care may, therefore, be underestimated. Unrecorded talk therapy by the GP may be skewed towards persons with poor income because GP consultations are free of charge for Danish patients, whereas there is a user fee of 40% for therapy by a psychologist after referral from GP. Still, unrecorded visits to psychologists may more likely be skewed towards persons of better socioeconomic status as they more often have private health insurance, or they can afford to pay a psychologist without referral from the GP despite a user fee of 100% (Gundgaard, 2006, Simon et al., 1996). Hence, unrecorded MH care may occur both among participants with lower socioeconomic status and among participants with higher socioeconomic status.

Another limitation is that we cannot be sure that the participant's MH care within the last year is related to the present episode of poor MH measured at the health check. SF-12 assesses MH status within the last four weeks. It would not seem advantageous to obtain data on MH care within the same time frame as MH care can occur with large time gaps and still be related to the actual occurrence of poor MH. We found it reasonable to extend the observation period to one year in order to capture most of the MH care related to the present poor MH status. This choice inevitably means that some MH care was related to previous, and not to the present, episode of poor MH. The extent of MH care is thus most likely an overestimation, and the true proportion of unaddressed MH problems may be greater.

### 4.3 Comparison with other studies

To our knowledge, only few other studies have included MH as a part of the risk assessment in a health check of the general population (Bjerkeset et al., 2006, Crisp and Priest, 1971). However, some studies have reported MH status in the baseline characteristics of participants in health checks of the general population (Hildrum et al., 2007, Chang et al., 2013, Gil et al., 2006, Jørgensen et al., 2009).

#### 4.3.1 Characteristics of health check participants with poor mental health

Other studies on health check participants have found similar associations with mental distress: female gender (Bjerkeset et al., 2006, Crisp and Priest, 1971, Hildrum et al., 2007, Gil et al., 2006, Jørgensen et al., 2009), low education (Bjerkeset et al., 2006, Hildrum et al., 2007, Gil et al., 2006, Jørgensen et al., 2009), unemployment (Bjerkeset et al., 2006, Jørgensen et al., 2009), risky health behaviour (Pisinger et al., 2009, Bjerkeset et al., 2006, Hildrum et al., 2007), and high risk for development of ischemic heart disease (Jørgensen et al., 2009). Although the age groups and the tools for assessing MH in these studies differed from the ones in *Check Your Health*, the results support our findings: Poor MH found in health check participants is associated with disadvantaged socioeconomy, health behaviour, and high risk of CVD. Similar associations between poor MH measured with the MCS of SF-12 and socioeconomic and health variables are also seen in the general Danish population (Christensen et al., 2014). This could indicate that MH assessment in a health check has the potential for reducing social inequality in MH. However, the proportion of poor MH among approximately same-aged persons (25–44 years) in the general population in a national Danish survey from 2013 was considerably larger (11.5%) (Christensen et al., 2014) than the proportion of poor MH in the health check (8.8%). This indicates that persons with poor MH are less likely to attend a health check.

#### 4.3.2 Potential for early intervention

Only 45% of participants identified with poor MH had already received MH care. This is in line with a meta-analysis, which showed that 47% of patients with major depression were recognised by the GP (Mitchell et al., 2009). General practice is the main provider of MH services (Bijl and Ravelli, 2000). Although persons with poor MH seem less likely to attend a health check, it seemed that a health check, after all, could reach persons with no or little contact to their own GP: Four out of five of those with apparently unacknowledged poor MH had seen their GP only once or not at all during the one year before the health check; this support our hypothesis: a health check might hold a potential for early intervention on MH problems.

#### 4.3.3 Social inequality in mental health care

Our findings of higher proportions of low socioeconomic status among participants who had received MH care were in contrast to other studies on the general population, including a Danish study that found lower use of MH services among persons of low socioeconomic status (Packness et al., 2017, Bijl and Ravelli, 2000, Have et al., 2003, Jokela et al., 2013, Hansen and Høye, 2015). However, links between socioeconomic factors and MH care in Europe are inconsistent in the literature. Some studies found higher use of MH services among persons of low socioeconomic status (Packness et al., 2017, Bijl and Ravelli, 2000, Have et al., 2003, Jokela et al., 2013, Aro et al., 1995, Ten Have et al., 2013, Davidsen et al., 2016), whereas others found no differences (Hansen and Høye, 2015, Ten Have et al., 2013). The studies are hard to compare due to methodological differences in applied socioeconomic parameters, MH care, MH status, and variations in the access to MH care in the different countries. As we did not have access to MH status on non-participants, we cannot be sure that our findings reflect that the GPs offer MH care to patients in highest need, including the socioeconomic disadvantaged groups. However, persons with low socioeconomic status are less likely to participate in the health check, and the health check attenders have a higher use of preventive services (Bjerregaard et al., 2017). Hence, our result may also be explained by a bias caused by a surplus of mental resources among the socioeconomic disadvantaged persons with poor MH who attended a health check compared to those that did not attend. Their participation in the health check may thus reflect stronger help-seeking behaviour in general.

## 5 Conclusion

Assessing the generic MH status in a health promoting program may hold a potential for improving MH. A total of 8.8% of health check participants were identified with poor MH with the use of the MCS of SF-12. They were more disadvantaged in terms of socioeconomic status and health status than those identified with moderate or good MH. More than half (55%) of participants identified with poor MH had not received any MH care as recorded in national health registers during the year before the health check. The proportions of participants with disadvantaged socioeconomic characteristics were high (43–55%) among those with (presumably) unrecognised MH problems.

Further studies are needed on how a coherent health system can best provide support for, not only identify, persons with poor MH. Additionally, we need more knowledge on which healthcare services may best target both mental and physical challenges.

## Conflict of interest

None.

## Funding

This work was founded by the Danish foundation TrygFonden, Central Denmark Region Foundation for Primary Health Care Research, the Health Foundation, the Danish General Practice Fund, Department of Public Health at Aarhus University, Graduate School of Health at Aarhus University, Danish Psychiatric Association, A.P. Møller Foundation for the Advancement of Medical Science, Hede Nielsen Family Foundation, Torben and Alice Frimodt's Foundation, and Christian and Ottilia Brorson's travel grant.

## Author contributions

CG, HTM, KC, EMD, and AS all contributed with developing the design of the study, interpretation of the work and critical revision of the scientific paper. CG is the guarantor of this work and accepts full responsibility for the conduct of the study; she had access to the data and controlled the decision to publish.
